# Differential associations of horizontally and vertically transmitted symbionts on *Ixodes ricinus* behaviour and physiology

**DOI:** 10.1186/s13071-023-06025-3

**Published:** 2023-11-29

**Authors:** Julian W. Bakker, Hannah L. M. Begemann, Manoj Fonville, Helen J. Esser, Willem F. de Boer, Hein Sprong, Constantianus J. M. Koenraadt

**Affiliations:** 1 Laboratory of Entomology, Wageningen University and Research, Wageningen, The Netherlands; 2 Centre for Infectious Disease Control, National Institute of Public Health and the Environment (RIVM), Bilthoven, The Netherlands; 3 Wildlife Ecology and Conservation Group, Wageningen University & Research, Wageningen, The Netherlands

**Keywords:** Ticks, *Midichloria mitochondrii*, *Borrelia burgdorferi*, Microbiome, Tick-borne pathogens, Horizontal transmission, Vertical transmission

## Abstract

**Background:**

*Ixodes ricinus* ticks are infected with a large diversity of vertically and horizontally transmitted symbionts. While horizontally transmitted symbionts rely on a vertebrate host for their transmission, vertically transmitted symbionts rely more on the survival of their invertebrate host for transmission. We therefore hypothesized horizontally transmitted symbionts to be associated with increased tick activity to increase host contact rate and vertically transmitted symbionts to be associated with higher tick weight and lipid fraction to promote tick survival.

**Methods:**

We used a behavioural assay to record the questing activity of *I. ricinus* ticks. In addition, we measured weight and lipid fraction and determined the presence of ten symbiont species in these ticks using qPCR, of which six were vertically transmitted and four horizontally transmitted.

**Results:**

Vertically transmitted symbionts (e.g. *Midichloria mitochondrii*) were associated with an increase in tick weight, whereas horizontally transmitted symbionts (e.g. *Borrelia burgdorferi* sensu lato) were often associated with lower weight and lipid fraction of ticks. Moreover, horizontally transmitted symbionts (e.g. *B. burgdorferi* s.l.) were associated with increased tick activity, which may benefit pathogen transmission and increases tick-borne disease hazard.

**Conclusions:**

Our study shows that horizontally and vertically transmitted symbionts differentially influence the behaviour and physiology of *I. ricinus* and warrants future research to study the underlying mechanisms and effects on transmission dynamics of tick-borne pathogens.

**Graphical abstract:**

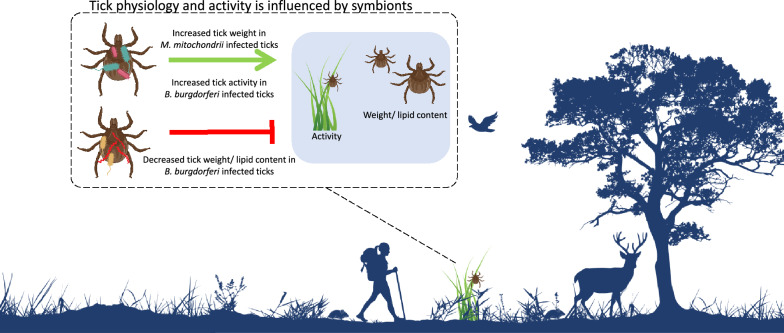

**Supplementary Information:**

The online version contains supplementary material available at 10.1186/s13071-023-06025-3.

## Introduction

The microbiome of ticks comprises a plethora of symbionts that can be transmitted horizontally to their vertebrate hosts or vertically to their offspring [1]. A further distinction can be made between symbionts that are pathogenic to their human host and symbionts that do not cause disease in humans [2]. These symbionts all have in common that they replicate in their tick vector. Whether their relationship with ticks is parasitic, commensal or mutualistic is not always clear. Furthermore, their impact on tick physiology and behaviour is poorly understood [3]. Therefore, a better understanding of the interactions between ticks and their symbionts would help to identify the mechanisms influencing the fitness of ticks and the effect of symbionts on the transmission of pathogens such as *Borrelia burgdorferi* sensu lato, the causative agents of Lyme disease.

Ticks have a long off-host period in which their primary energy source comes from their blood meal obtained in the previous life stage. This blood is converted to energy sources such as lipids, glycogen and carbohydrates [4]. Throughout the off-host period, these energy reserves are depleted when ticks are questing for a host [5, 6]. Furthermore, unfavourable conditions for tick survival, such as low humidity or high temperatures, increase lipid metabolism [7]. The behavioural choices that ticks make during the off-host period may therefore influence the survival of ticks and consequently the transmission of pathogenic symbionts to vertebrate hosts.

Human pathogenic symbionts that are transmitted horizontally, such as *B. burgdorferi* s.l. and tick-borne encephalitis virus (TBEV), may benefit from a high questing activity of ticks and could manipulate ticks to become more active, thereby increasing their chances of being transmitted to a host [8]. Examples of symbionts that influence the behaviour and physiology of their vector to advance their own dispersal are many. For example, baculoviruses increase the climbing behaviour and locomotion of their lepidopteran hosts, thereby promoting their spread to other caterpillars [9]. *Plasmodium falciparum*, the protozoan parasite causing human malaria, manipulates mosquito feeding behaviour by enhancing the attraction to human odour [10]. In ticks, *B. burgdorferi* s.l. has been associated with increased activity and lipid fraction of the tick vector *Ixodes ricinus* [11]. *Anaplasma phagocytophilum* infection induces the expression of antifreeze genes protecting ticks under cold conditions [12], and *Babesia* infection in rodents increases the engorgement weight and moulting success of *Ixodes trianguliceps* ticks [13]. Most studies on the effects of symbionts on tick behaviour and physiology have focussed on symbionts that are pathogenic to humans [14]. Relatively little is known about the effect of symbionts, such as *Rickettsia helvetica, Midichloria mitochondrii*, *Spiroplasma ixodetis* and *Rickettsiella* spp., on tick physiology and behaviour.

The relationship between *Ixodes* ticks and some of their symbionts has probably evolved from the obligate haematophagous feeding behaviour of ticks, as this diet lacks essential nutrients such as B vitamins [3, 12], which are important for the development of arthropods [15]. The microbiome of *Ixodes* ticks is dominated by symbionts such as *Francissella* that can produce B vitamins such as biotin, riboflavin and folate [12, 16]. Besides providing essential nutrients, tick symbionts reduce the time to oviposition and increase larval hatching success [12]. These intricate interactions between ticks and their symbionts suggest that the survival, behaviour and physiology of ticks depend on the presence of certain symbionts.

In this study, we aimed to investigate the effects of vertically and horizontally transmitted *I. ricinus* symbionts on the physiology and behaviour of their tick vector (Table 1). We used a behavioural assay to track and quantify tick movement. In addition, we determined the weight and lipid fraction of ticks to understand the associations between symbionts and the physiology of ticks. We expected horizontally transmitted symbionts to be associated with differences in tick behaviour and vertically transmitted symbionts to be associated with differences in tick physiology. Specifically, as increased host-contact rates are beneficial for horizontally transmitted symbionts such as *B. burgdorferi* s.l. and *Neoehrlichia mikurensis* [29], we expected increased activity of ticks infected with these symbionts. In contrast, vertically transmitted symbionts may promote the survival of their host, and hence their own transmission, by supporting larger blood meals and thus increased energy resources. We therefore expected to find higher body weights and lipid fractions in ticks with vertically transmitted symbionts.

**Table 1:** Symbionts in this study and their transmission mode

Symbiont	Transmission mode	References
*Anaplasma phagocytophilum*	Horizontal	[17]
*Neoehrlichia mikurensis*	Horizontal	[18]
*Babesia microti* (clade I)	Horizontal	[19]
*Borrelia burgdorferi* s.l	Horizontal	[20]
*Borrelia miyamotoi*	Vertical	[21]
*Rickettsia helvetica*	Vertical	[22]
*Rickettsiella* spp.	Vertical	[23]
*Babesia* spp. (clade X)^a^	Vertical	[24, 25]
*Spiroplasma ixodetis*	Vertical	[26]
*Midichloria mitochondrii*	Vertical	[27]

^a^*Babesia* spp. from clade X, which have been found in (Dutch) *I. ricinus* are *Babesia divergens, B. venatorum, B. capreoli* and *B. odocolei*-like [28]

## Materials and methods

### Tick collection

Between February and August 2021, ticks were collected by dragging a 1 m^2^ white cotton cloth over the vegetation of a mixed forest plot at the Dorschkamp (51°58′37.3″N 5°41′57.8″E, Wageningen, The Netherlands). Ticks of the nymphal life stage were collected per 25 individuals and stored in 15-ml Falcon tubes. Three different groups of ticks were used during this study. A first group of 728 ticks were used to study the associations among symbionts, tick weight and lipid fraction. These ticks were immediately frozen at – 20 °C on the day of collection. A second group of 607 ticks was used to study the associations between symbionts and tick activity in a tick behaviour assay. In contrast with the first group, these ticks were not weighed or analysed for lipid content but were kept alive by storing them at ~ 90% RH in an incubator set at 18:6 L:D and 18 °C for up to 1 month for the activity experiment. These ticks were frozen at − 20 °C after the activity experiment and used for subsequent pathogen analyses. A third experiment was carried out to confirm the observed associations from the previous two experiments, but now we investigated the associations between symbionts, tick weight, lipid fraction and tick activity simultaneously in one group of 165 ticks. Ticks were stored at ~ 90% RH in an incubator set at 18:6 L:D and 6 °C for up to 2 weeks for the activity experiment. These ticks were frozen at − 20 °C after the activity experiment and used for subsequent pathogen, weight and lipid analyses.

### Weight and lipid analysis of nymphs

Individual nymphs of the first and third tick group were dried overnight at 70 °C before weighing them twice using a microbalance (Sartorius, Germany) [30]. The average dry weight of the two measurements was used for analysis. After weighing, the lipid content was determined using a protocol adapted from Alasmari and Wall [7] and Abdullah and colleagues [6]. Individual ticks were then homogenized using a pestle and liquid nitrogen. Next, 100 μl sterile Milli-Q H_2_0 was added, samples were mixed, and an aliquot of 45 μl was removed and frozen for later symbiont analysis. The remainder of the sample was used for lipid analyses. To this, 200 μl of a 2% sodium sulphate solution (VWR International, Leicestershire, UK) and 860 μl of a 1:1 chloroform–methanol mixture were added. Together with a chloroform blank, a lipid standard dilution series was made in triplicate in 1.5-ml Eppendorf tubes with soybean oil dissolved in a 1:1 chloroform–methanol mixture (0.98 mg/ml). Lipid standards ranged from 5 to 40 μg. To these tubes, the same amounts of sodium-sulphate solution and a 1:1 chloroform–methanol mixture were added. Tubes were then vortexed and centrifuged for 5 min at 179* g* at 4 °C, after which 550 μl was removed and pipetted in borosilicate glass tubes (12 × 75 × 1 mm). Liquid was evaporated by placing tubes in a 90 °C water bath. To break down the lipid strands, 100 μl of 95% sulphuric acid was added, and tubes were vortexed and incubated in the water bath at 90 °C for 15 min. The reaction was stopped by putting samples on ice. Subsequently, 1 ml of a 1.2 g/l vanillin solution (Sigma-Aldrich, St Louis, MO) in 68% phosphoric acid (Sigma-Aldrich) was added. Tubes were vortexed and incubated for 15 min at room temperature. Then, 100 μl of this mix was pipetted in a 96-well plate, and absorbance was measured at 525 nm with a spectrophotometer (Microplate Spectrophotometer Multiskan Sky; Thermo Scientific, Waltham, MA).

Based on the absorbance of the standard dilution series, a standard curve was made to determine the lipid content of the actual tick samples. As larger ticks had higher amounts of lipids (Additional file 1: Fig. S1A and S1B), the lipid amount was expressed as a fraction by dividing the lipid weight by the tick dry weight [30]. As we used an adapted protocol for lipid determination, we conducted a pilot study to show that we were able to reliably determine differences in lipid fraction and weight of ticks after exposure to different temperatures for 2 weeks (Additional file 1: Fig S2A and S2B).

### Tick behaviour assay

Tick questing behaviour was investigated using a set-up (Additional file 1: Fig. S3A) consisting of 29 vertical transparent polycarbonate tubes (2 × 12 × 450 mm) with mesh on both sides to prevent ticks from escaping while allowing airflow. Ticks could walk freely up and down inside these polycarbonate tubes. The tubes were placed in a terrarium (600 × 400 × 500 mm) with a 2-cm water layer at the bottom to create a humidity gradient. This water layer was kept at a constant temperature of 10 °C by pumping water through copper tubing using an RTE-100 Refrigerated Bath Circulator (Thermo Neslab, Waltham, MA) [31]. Besides a constant temperature at the bottom, the water circulation also ensured a vertical relative humidity gradient. Illumination was provided using two LED panels (600 × 600 mm, 40W, 4000 lm, 6500 Kelvin, Ledvance, Capelle aan den Ijssel, The Netherlands). Temperature and relative humidity (RH) at the bottom of each tube and of the environment were measured before and after each run using data loggers with external probes (MSR Electronics GmbH, Henngard, Switzerland). Researchers wore gloves when handling tubes and ticks in the bioassay, and the bioassay was cleaned after each run using 15% ethanol.

Ticks were placed individually at the bottom of a tube at the start of each run. Ticks were filmed for 7 h using a Basler acA1920-155um camera (Basler, Ahrensburg, Germany) with a 16-mm f/1.4 Canon lens and recorded using Media Recorder 4 software (Noldus, Wageningen, The Netherlands). After each run, ticks were collected and stored at – 20 °C until weight, lipid or symbiont analyses. Tracking files were analysed using Ethovision XT (version 16, Noldus, Wageningen, The Netherlands). Velocity, total distance walked, maximum and mean questing height and the proportion of time spent in either the low or high humidity zone were calculated. Low and high humidity zones in the tubes were defined based on the saturation deficit (SD), which influences the questing behaviour of ticks [29]. The SD was calculated according to Perret et al. (2000). An SD < 5.6 was defined as the high humidity zone (> 68% RH) and an SD > 9 was defined as the low humidity zone (< 50% RH, Additional file 1: figure S3B).

### DNA extraction and symbiont detection

DNA was extracted from individual ticks by alkaline lysis using ammonium hydroxide as described by Wielinga and colleagues [32], and lysates were stored at 4 °C until analysis. Ticks were tested with qPCR for the presence of the horizontally transmitted *B. burgdorferi* s.l. [33], *Anaplasma* spp. [34]*, N. mikurensis* [35] and *Babesia microti* [26] and the vertically transmitted *Borrelia miyamotoi* [36]*, R. helvetica* [37]*, Rickettsiella* spp. [38]*, Babesia* spp. [39], *S. ixodetis* [26] and *M. mitochondrii* [38]*.* We did not test for the presence of *Coxiella*-like or *Francisella*-like symbionts in this study, as these have not been found in *I. ricinus* in The Netherlands before [38].

### Statistical analysis

Various statistical models were used to study associations of symbionts with the weight or lipid fraction of ticks. Models included weight or lipid fraction as dependent variable and either the individual symbiont species or symbionts pooled by their transmission mode (horizontally or vertically) as main effects. We further analysed the impact of symbiont-symbiont interactions by including the interaction term of the two symbionts as main effects. The weight of the tick was included as a covariate when analysing the lipid fraction. The day on which the ticks were collected was included as a nominal random factor as ticks were collected between April and August on 9 different days. Models for tick weight were analysed using linear mixed models (LMMs) with a Gaussian distribution and identity link function, whereas models for lipid fraction were analysed using generalized linear mixed models (GLMMs) with a gamma distribution and log link function.

Principal component analysis (PCA) was used to further investigate the variables describing the behaviour of ticks in the behavioural assay [40]. Average questing height, maximum questing height, time spent in the high humidity zone or the low humidity zone, velocity (mm/s) and distance moved (mm) were included in the PCA. The Kaiser-Guttman criterion was then used to reduce the number of principal components to retain based on an eigenvalue > 1 [41]. This resulted in two principal components that each explained different aspects of tick behaviour. The associations of tick behaviour with symbionts were assessed using the two principal components as dependent variables in separate generalized linear models (GLMs) with a Gaussian distribution and log link function. The principal components were transformed by adding a constant to the data (Y + 5) to account for negative values that could not be analysed using a log link function [42]. The symbionts (individual, pooled per transmission route or the interaction between two groups) were included as main effects. In addition, the day of collection (5 days between February and April 2021) and time of the day an assay was run (morning or afternoon) were also included as main effects (i.e. nominal predictors) because the number of levels was lower than the recommended number of levels for inclusion as random effects.

Linear models (LMs) were used with a Gaussian distribution and identity link function for the third (smaller) group of ticks for which we assessed both tick behaviour and lipid fraction and weight. Tick weight, lipid fraction, symbiont (individual, pooled per transmission route or the interaction between two groups) were included as main effects. Furthermore, the collection day (5 days between April and May) was included as main effect (nominal predictors) as the number of levels was lower than the recommended number of levels for inclusion as random effects.

For single symbiont and co-infection analyses, symbionts with an infection prevalence of < 2% were not included in the analyses. All statistical analyses were carried out using the *glmmTMB* package in R [43]. Likelihood ratio or Wald *χ*^2^ tests were used to test the significance of main effects in the models. Post hoc pairwise comparisons were made using the *emmeans* package [44] in R (Version 4.2.0). The Akaike information criterion (AIC) was used for model selection [45].

Co-occurrence of tick symbionts was tested using the *co-occur* package [46, 47]. This package uses a hypergeometric distribution and presence-absence data to calculate the probability that a tick is co-infected with two symbionts and whether this co-infection occurs more or less frequently than expected.

## Results

### Symbiont prevalence in *Ixodes ricinus*

**Fig. 1:**
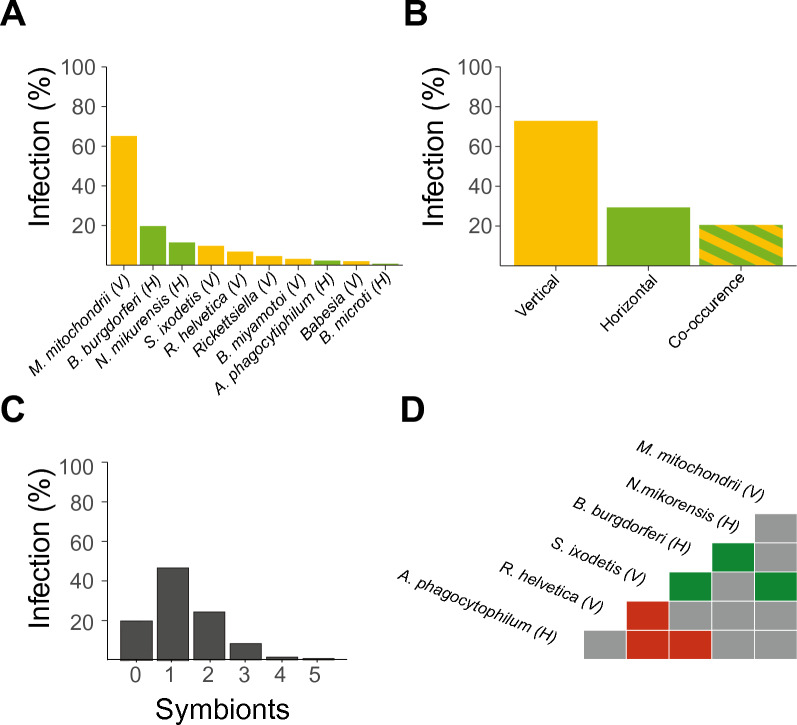
**A** Infection percentage of individual ticks with each of the 10 symbiont species. **B** Infection percentage of either vertically transmitted symbionts, horizontally transmitted symbionts or co-infection with a horizontally and a vertically transmitted symbiont. **C** Infection percentages of the number of horizontally and vertically transmitted symbionts combined per nymph. **D** Co-occurrence matrix of symbionts. Only significant co-occurrences are shown. Green and red colours indicate significant (*P* < 0.05) positive or negative co-occurrence of the symbiont species in ticks, respectively. The transmission mode is depicted as (V) vertically or (H) horizontally

A total of 1500 ticks were collected and tested for the presence of 10 different symbiont species (Table 2**, **Fig. 1A). The vertically transmitted *M. mitochondrii* and horizontally transmitted *B. burgdorferi* s.l. were the most abundant symbiont species in the *I. ricinus* nymphs with an infection prevalence of 65.5% and 19.8% respectively.

**Table 2:** Infection prevalence (%) of symbionts per experimental group

	Total (n)	*B. burgdorferi* s.l. (H) (%)	*N. mikurensis *(H) (%)	*A. phagocytophilum *(H) (%)	*B. microti* (H) (%)	*M. mitochondrii* (V) (%)
Physiology	728	22.9	13.4	1.6	0.3	70.4
Activity	607	16.3	8.8	2.9	0.3	61.8
Physiology and activity	165	14.2	12.4	1.8	0.6	57.4
Total	1500	19.8	11.5	2.2	0.3	65.5

	Total (n)	*Babesia* spp. (V) (%)	*R. helvetica* (V) (%)	*Rickettsiella* spp. (V) (%)	*B. miyamotoi* (V) (%)	*S. ixodetis* (V) (%)
Physiology	728	2.9	5.3	0.8	4.2	10.3
Activity	607	1.2	8.6	10.1	2.5	9.3
Physiology and activity	165	1.2	7.7	1.8	1.2	7.1
Total	1500	2.0	6.9	4.7	3.2	9.5

H: Symbionts that are primarily horizontally transmitted; V: symbionts that are primarily vertically transmitted

At least one vertically transmitted symbiont species was present in 72.9% of the collected nymphal ticks, whereas horizontally transmitted symbiont species were present in 29.3% of the nymphs (Fig. 1B). Vertically and horizontally transmitted symbionts co-occurred in 21.8% of the nymphs (Fig. 1B). Of all the nymphs tested, 0.1% were infected with five symbiont species, 1.4% were infected with four symbiont species, 8.2% were infected with three symbiont species, 24.2% were infected with two symbiont species, and approximately half of the nymphs (46.4%) were infected with one symbiont species (Fig. 1C). About one in five (19.7%) nymphs was not infected with any of the ten symbiont species for which we tested.

Significant negative co-occurrences were observed for *A. phagocytophilum* with *S. ixodetis* and *B. burgdorferi* s.l., and for *R. helvetica* with *S. ixodetis* (Fig. 1D, Additional file 1: Table S1). In addition, positive co-occurrences were observed for *B. burgdorferi* s.l. with *N. mikurensis* and for *S. ixodetis* with *M. mitochondrii* and with *B. burgdorferi* s.l. No significant co-occurrences were observed for the other symbiont-symbiont combinations.

### Tick weight and lipid fraction

**Fig. 2:**
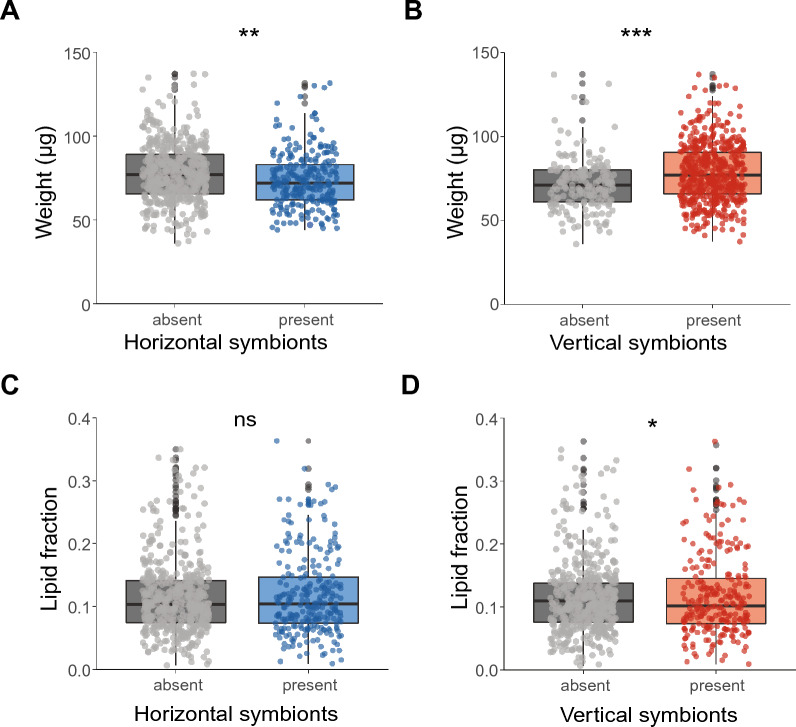
Associations between weight or lipid fraction and the presence or absence of horizontally and vertically transmitted symbiont species. **A** Weight (µg) of ticks with or without horizontally transmitted symbionts. **B** Weight (µg) of ticks with or without vertically transmitted symbionts. **C** Lipid fraction of ticks with or without horizontally transmitted symbionts. **D** Lipid fraction of ticks with or without vertically transmitted symbionts. The lipid fraction was calculated by dividing the lipid weight by the tick dry weight. GLM: ns = not significant, *P < 0.05, **P < 0.01, ***P < 0.001

For the first group of individual ticks, the weight and lipid fraction of ticks were determined to study the associations between the presence or absence of symbiont species and tick physiology. When symbiont species were pooled by their transmission mode, the presence of the vertically transmitted symbionts was associated with a higher median body weight of ticks of 8.5% (GLMM, *χ*^2^ = 28.1, df = 1, *P* < 0.001, Fig. 2B), which is in agreement with our hypothesis. In contrast, the presence of the horizontally transmitted symbionts was associated with a 7% lower median body weight of ticks (GLMM, *χ*^2^ = 9.0, df = 1, *P* < 0.01, Fig. 2A). In general, ticks with a higher lipid fraction were also heavier (GLMM, *χ*^2^ = 16.9, df = 1, *P* < 0.001). The presence of horizontally transmitted symbionts was not associated with the lipid fraction of ticks (GLMM, *χ*^2^ = 2.4, df = 1, *P* > 0.05, Fig. 2C), whereas the presence of vertically transmitted symbionts was associated with a reduced median lipid fraction of 7.6% of the ticks (GLMM, *χ*^2^ = 4.5, df = 1, *P* < 0.05, Fig. 2D), which contradicts our hypothesis. We observed a significant random effect of day on which the ticks were collected for all (G)LMMs used.

**Fig. 3:**
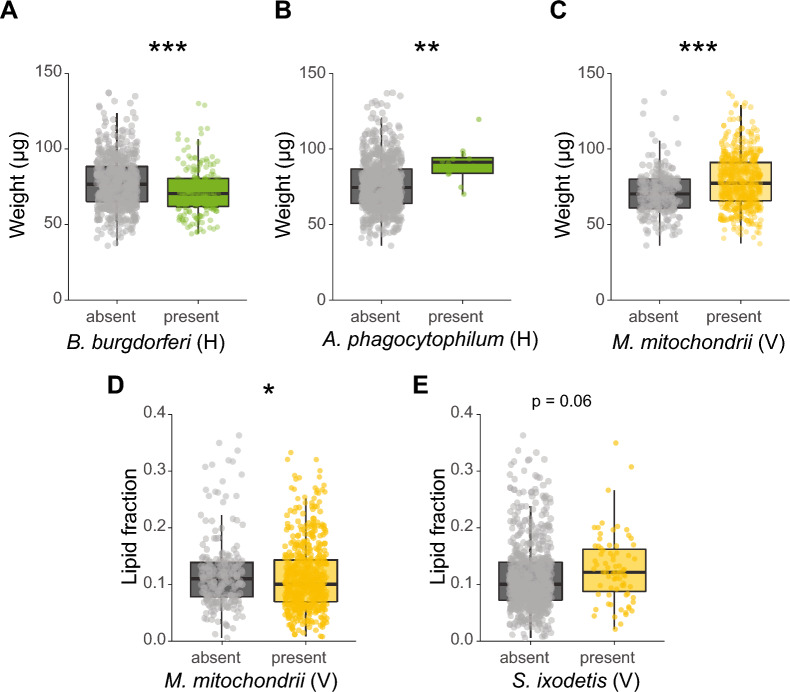
Associations of weight or lipid fraction and the presence or absence of specific symbiont species. Only significant associations of weight or lipid fraction with symbionts are shown in the graphs. **A** Weight (µg) of *Borrelia burgdorferi* s.l. (un)-infected ticks. **B** Weight (µg) of *Anaplasma phagocytophilum* (un)-infected ticks. **C** Weight (µg) of *Midichloria mitochondrii* (un)-infected ticks. **D** Lipid fraction of *M. mitochondrii* (un)-infected ticks. **E** Lipid fraction of *Spiroplasma ixodetis* (un)-infected ticks. The lipid fraction was calculated by dividing the lipid weight by the tick dry weight. The transmission mode is depicted as (V) vertically or (H) horizontally. GLM: *P < 0.05, **P < 0.01, ***P < 0.001

At the individual symbiont species level, two horizontally transmitted symbionts were significantly associated with the weight of ticks: *B. burgdorferi* s.l.-infected ticks had an 8.8% lower median body weight (GLMM, *χ*^2^ = 12.9, df = 1, *P* < 0.001, Fig. 3A), whereas *A. phagocytophilum*-infected ticks had a 22.5% higher median body weight (GLMM, *χ*^2^ = 7.2, *P* < 0.01, Fig. 3B) compared to uninfected ticks. Of note, infection with *A. phagocytophilum* was observed in 12 (1.6%) ticks, whereas infection with *B. burgdorferi* s.l. was observed in 176 (22.9%) ticks. Consistent with the analyses in which symbionts were pooled according to their transmission mode, no association was observed between the presence of horizontally transmitted symbiont species and the lipid fraction of nymphs.

Of the vertically transmitted symbiont species, only ticks infected with *M. mitochondrii* had a 10.3% higher median body weight (LRT, *χ*^2^ = 39.7, df = 1, *P* < 0.001, Fig. 3C). Furthermore, *M. mitochondrii*-infected ticks had a 9.9% lower median lipid fraction compared to uninfected ticks (GLMM, *χ*^2^ = 5.2, df = 1, *P* < 0.05, Fig. 3D). Ticks infected with the vertically transmitted *S. ixodetis* had a 19.8% higher median lipid fraction, but this difference was not significant (GLMM, *χ*^2^ = 3.4, df = 1, *P* = 0.06, Fig. 3E).

**Fig. 4:**
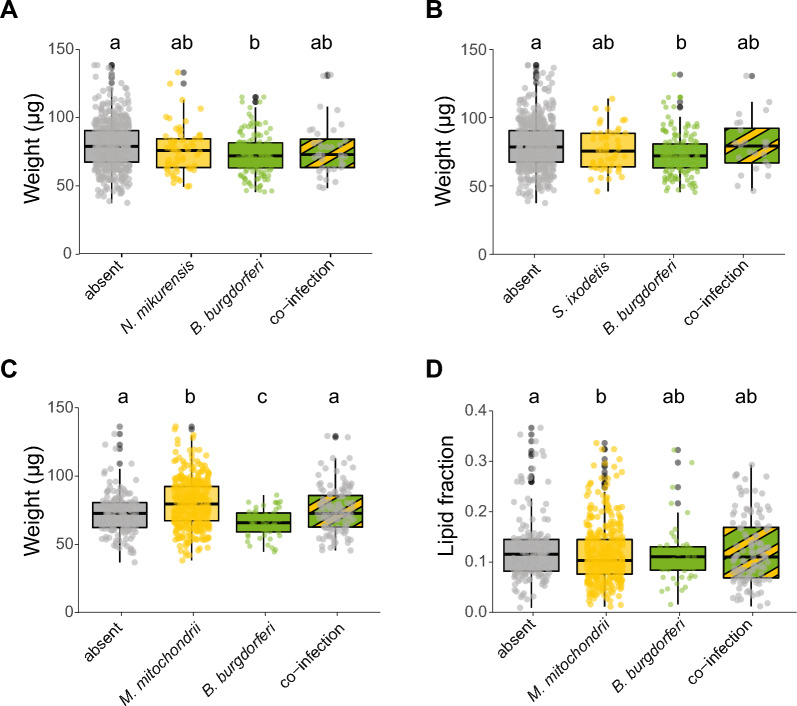
Associations of weight or lipid fraction and the presence or absence of co-infections of specific symbionts. **A** Weight (µg) of ticks single infected or co-infected with *Borrelia burgdorferi* s.l. and *Neoehrlichia mikurensis*. **B** Weight (µg) of ticks single infected or co-infected with *B. burgdorferi* s.l. and *Spiroplasma ixodetis*. **C** Weight (µg) of ticks single infected or co-infected with *B. burgdorferi* s.l. and *Midichloria mitochondrii*. **D** Lipid fraction of ticks single infected or co-infected with *B. burgdorferi* s.l. and *M. mitochondrii.* The lipid fraction was calculated by dividing the lipid weight by the tick dry weight. Boxplots within each panel that have no letters in common are significantly different (GLM, emmeans, *P* < 0.05)

Symbiont-symbiont interactions of the four most prevalent symbionts were further analysed to study the associations of co-infection status with tick weight and lipid fraction. These analyses included the two most common vertically transmitted species, *M. mitochondrii* and *S. ixodetis*, and the two most common horizontally transmitted species, *B. burgdorferi* s.l. and *N. mikurensis*, in our study (Fig. 1A). When analysing the relation between symbionts and tick weight, a statistically significant interaction between *B. burgdorferi* s.l. and *N. mikurensis* was observed (GLMM, LRT, *χ*^2^ = 3.8, df = 1, *P* < 0.05, Fig. 4A). This was also the case for the interaction between *B. burgdorferi* s.l. and *S. ixodetis* (GLMM, LRT, *χ*^2^ = 3.9, df = 1, *P* < 0.05, Fig. 4B). In both analyses, ticks infected with *B. burgdorferi* s.l. had a significantly lower weight compared to uninfected ticks (Fig. 4A and B). However, the presence of *N. mikurensis* or *S. ixodetis* cancelled out the negative effect of *B. burgdorferi* s.l. on tick weight. No statistically significant interaction was observed between *B. burgdorferi* s.l. and *M. mitochondrii* (GLMM, LRT, χ^2^ = 0.7, df = 1, *P* > 0.05), but the lower weight of *B. burgdorferi* s.l.-infected ticks or the higher weight of *M. mitochondrii*-infected ticks was cancelled out in co-infected ticks (Fig. 4C).

We further studied the associations between symbiont co-infection and the lipid fraction of ticks. A statistically significant interaction between *M. mitochondrii* and *B. burgdorferi* s.l. was observed (GLMM, LRT, *χ*^2^ = 5.1, df = 1, *P* < 0.05, Fig. 4D). Ticks infected only with *M. mitochondrii* had a significantly lower lipid fraction compared to uninfected ticks (Fig. 4D). However, the presence of *B. burgdorferi* s.l. in ticks cancelled out this association as co-infected ticks did not have significantly different lipid fractions compared to uninfected ticks.

### Behavioural analysis of ticks

**Fig. 5:**
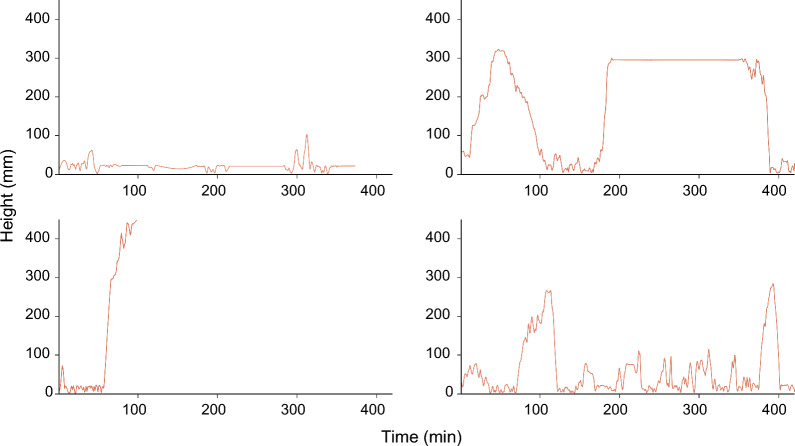
Examples of four activity patterns of four individual *Ixodes ricinus* nymphs. Each panel represents the activity pattern of a single nymph

Analyses of the second group of ticks showed distinct activity patterns among the individual ticks that were observed in the behavioural assay (Fig. 5). These activity patterns were subsequently used to derive several behavioural parameters. PCA was used to analyse the behaviour of ticks based on these multiple, possibly collinear behavioural parameters (e.g. velocity, distance, questing height etc.). The principal components were then used to study the associations between these behavioural parameters and presence of symbiont species.

**Fig. 6:**
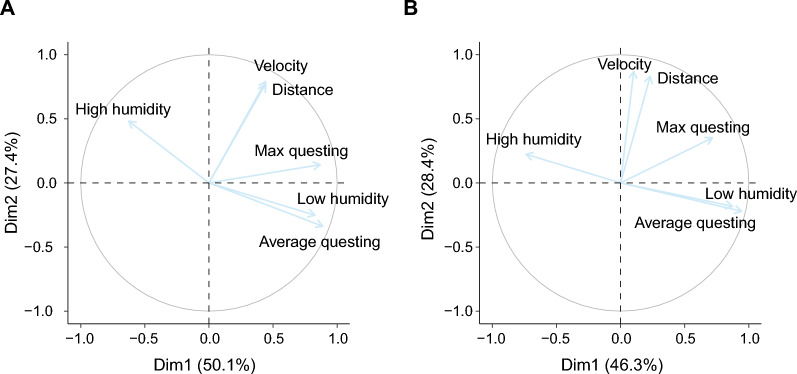
Biplot of the principal component analysis (PCA) of the behavioural parameters of ticks. **A** Biplot corresponding to the behavioural experiment of ticks (based on 607 individuals). **B** Biplot corresponding to the behaviour and physiology experiment of ticks (based on 165 individuals)

The six parameters associated with tick behaviour were reduced to two principal components, explaining respectively 50.1% and 27.4% of the variance (Table 3, Fig. 6A). The first component (PC1) axis scores were positively correlated with questing height (average questing height and maximum questing height) and the low humidity zone. The second component (PC2) axis scores were positively associated with walking distance and velocity and to a lesser extent with the high humidity zone.

**Table 3:** Coordinates and contributions of each of the behavioural variables to the first two principal components of the second experimental tick group

	PC1		PC2	
Variable	Coordinate	Contribution	Coordinate	Contribution
Average questing height	0.89	0.79	− 0.33	0.11
Maximum questing height	0.86	0.75	0.14	0.02
Low humidity zone	0.82	0.68	− 0.25	0.06
High humidity zone	− 0.62	0.39	0.48	0.23
Distance	0.43	0.19	0.77	0.59
Velocity	0.44	0.19	0.79	0.62
Eigenvalue	3.00		1.63	
Variance (%)	50.12		27.42	
Cumulative variance (%)	50.1		77.5

**Fig. 7:**
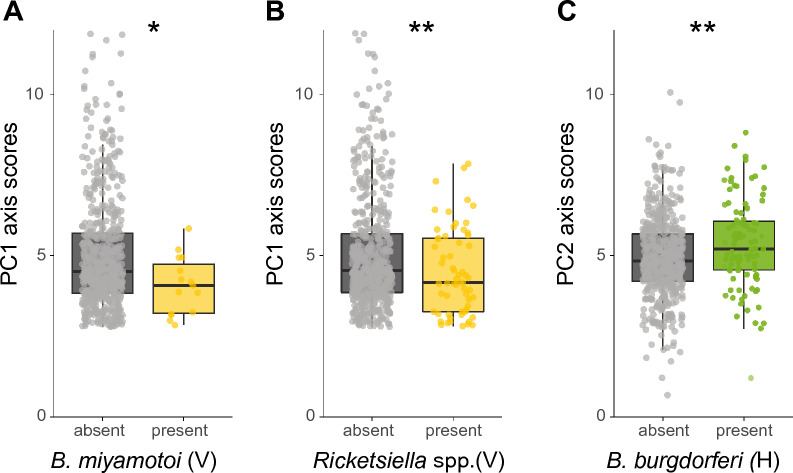
Associations of PC1 and PC2 axis scores with selected symbionts. **A** PC1 axis scores of *Borrelia miyamotoi* (un)-infected ticks. **B** PC1 axis scores of *Rickettsiella* spp. (un)-infected ticks. **C** PC2 axis scores of *Borrelia burgdorferi* s.l. (un)-infected ticks. The transmission mode is depicted as (V) vertically or (H) horizontally. GLM: *P < 0.05, **P < 0.01

The presence or absence of horizontally or vertically transmitted symbionts was not associated with either the PC1 or PC2 axis scores (GLM, *P* > 0.05 for both variables, Additional file 1: Table S2). Nevertheless, we observed symbiont-specific associations for both principal components. For the vertically transmitted symbionts, *B. miyamotoi* and *Rickettsiella* spp., infected ticks had lower PC1 axis scores (GLMM *χ*^2^ = 5.4, df = 1, *P* < 0.05 and GLM, *χ*^2^ = 6.7, df = 1, *P* < 0.01, respectively, Fig. 7A and B), indicating that ticks infected with these two vertically transmitted symbionts quested lower and spent less time in the low humidity zone (located at the highest point in the assay) compared to uninfected ticks. Of the horizontally transmitted symbiont species, *B. burgdorferi* s.l. infected ticks had higher PC2 axis scores (GLM, *χ*^2^ = 7.3, df = 1, *P* < 0.01, Fig. 7C), indicating that ticks infected with *B. burgdorferi* s.l. moved further and faster compared to uninfected ticks. All other individual symbiont species were not significantly associated with either PC1 or PC2 axis scores. There was a significant effect of the date at which ticks were collected for both the PC1 and PC2 axis scores analyses. No significant interactions were observed between symbiont co-infections and PC1 or PC2 axis scores.

### Tick behaviour and physiology combined

A third group of 165 ticks was used to study the associations among tick behaviour, physiology and the presence of symbiont species within the same experiment. Also here, PCA reduced the behavioural parameters to two components, explaining 46.3% and 28.37% of the variance respectively (Table 4**, **Fig. 6B). Similar to the second experiment, the questing height and humidity zones strongly correlated to PC1 axis scores, and the distance and velocity parameters correlated to PC2 axis scores.

**Table 4:** Coordinates and contributions of each of the behavioural variables to the first two principal components of the third experimental tick group

Variable	PC1		PC2	
	Coordinate	Contribution	Coordinate	Contribution
Average questing height	0.95	0.89	− 0.22	0.05
Maximal questing height	0.73	0.51	0.35	0.13
Low humidity zone	0.87	0.54	0.22	0.05
High humidity zone	− 0.74	0.76	0.87	0.03
Distance	0.23	0.05	0.83	0.69
Velocity	0.10	0.01	0.87	0.76
Eigenvalue	2.77		1.70	
Variance	46.27		28.37	
Cumulative variance %	46.27		74.64	

**Fig. 8:**
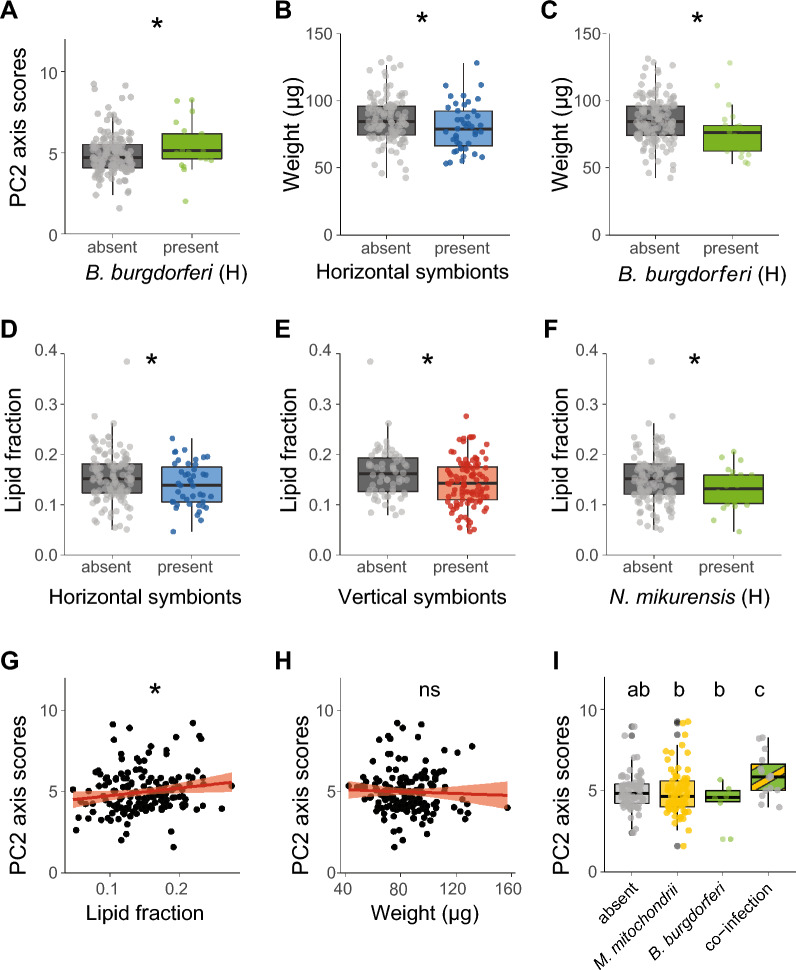
Associations among symbionts, behaviour and physiology of *Ixodes ricinus* nymphs. **A** PC2 axis scores of *Borrelia burgdorferi* s.l. (un)-infected ticks. **B** Weight (µg) of (un)-infected ticks with horizontally transmitted symbionts. **C** Weight (µg) of *B. burgdorferi* s.l. (un)-infected ticks. **D** Lipid fraction of (un)-infected ticks with horizontally transmitted symbionts. **E** Lipid fraction of (un)-infected ticks with vertically transmitted symbionts. **F** Lipid fraction of *Neoehrlichia mikurensis* (un)-infected ticks. **G** Association of PC2 axis scores with lipid fractions of ticks. The lipid fraction was expressed as the lipid weight divided by the tick dry weight. **H** Association of PC2 axis scores with tick weight (µg). **I** PC2 axis scores of ticks single infected or co-infected with *B. burgdorferi* s.l. and *Midichloria mitochondrii*. The lipid fraction was calculated by dividing the lipid weight by the tick dry weight. The horizontally transmission rmode is depicted as (**H**). GLM: ns = not significant, *P < 0.05. Boxplots within each panel that have no letters in common are significantly different (GLM, emmeans *P* < 0.05)

No associations were found between the presence of horizontally transmitted symbionts and PC1 axis scores. For PC2 axis scores, ticks infected with *B. burgdorferi* s.l. showed increased activity (distance moved, velocity) compared to uninfected ticks (GLM, *χ*^2^ = 4.1, df = 1, *P* < 0.05, Fig. 8A), as also observed in the second experiment. None of the vertically transmitted symbionts were associated with either score on PC1 or PC2 axes, as opposed to the previous experiment in which we observed associations of PC1 axis scores with *B. miyamotoi* and *Rickettsiella* spp.

As we determined the weight and lipid fraction of ticks in this third experiment, we could also infer associations among symbiont species, tick physiology and tick behaviour. When symbionts were pooled by their transmission mode, we observed a negative association between the presence of horizontally transmitted symbionts and tick weight (GLM, χ^2^ = 5.0, df = 1, *P* < 0.05, Fig. 8B), as in the first experiment. Moreover, we also confirmed our previous findings at the symbiont species level that *B. burgdorferi* s.l.-infected ticks had a significantly lower weight compared to uninfected ticks (GLM, *χ*^2^ = 4. 8, df = 1, *P* < 0.05, Fig. 8C). The lipid fraction was lower in ticks infected with horizontally or vertically transmitted symbionts (GLM, *χ*^2^ = 4.2, df = 1, *P* < 0.05, GLM, *χ*^2^ = 4.1, df = 1, *P* < 0.05, respectively, Fig. 8D and E**)**. At the symbiont species level, however, only ticks infected with *N. mikurensis* had a lower lipid fraction (GLM, *χ*^2^ = 4.8, df = 1, *P* < 0.05, Fig. 8F).

In contrast to the first two experiments, we could also investigate associations between tick weight and lipid fraction and the behavioural parameters (PC1 and PC2 scores) in the third experiment. The weight and lipid fraction of ticks was not associated with PC1 axis scores. The lipid fraction of ticks was positively associated with PC2 axis scores (*χ*^2^ = 5.9, df = 1, *P* < 0.05, Fig. 8G), whereas the weight of ticks was not associated with PC2 axis scores (Fig. 8H). This suggests that the distance travelled and walking velocity of ticks are positively associated with higher lipid fractions.

We further tested the associations of co-infections of the four most prevalent symbionts as described above. Of the six possible combinations, only the statistical interactions between *B. burgdorferi* s.l. and *M. mitochondrii* had significantly different PC2 axis scores (*χ*^2^ = 6.3, df = 1, *P* < 0.05, Fig. 8I). Specifically, co-infection of *B. burgdorferi* s.l. with *M. mitochondrii* was associated with increased PC2 axis scores compared to single infection with either symbiont or absence of both symbionts, suggesting that co-infection of these two symbionts increases the walking distance and velocity of ticks.

Summarizing all available results, we identified consistent associations between horizontally transmitted symbionts, in particular for *B. burgdorferi* s.l., with tick behaviour and physiology (Table 5). Infection with this symbiont was associated with reduced weight and increased activity in all experiments. For the vertically transmitted symbionts, we detected inconsistent associations with weight but consistent associations with lipid fraction (Table 5). More specifically, *M. mitochondrii* infection was associated with higher weight and reduced lipid fraction only in one of the two experiments. Symbiont-specific observations were observed for *N. mikurensis, M. mitochondrii*, *A. phagocytophilum*, *B. miyamotoi* and *Rickettsiella* spp. that were not consistent among experiments (Table 5). These discrepancies were most likely related to different sample sizes and small effect sizes of the observed associations.

**Table 5:** Overview of observed associations between symbionts and physiological or behavioural parameters from this study

Experiment	Tick weight			Lipid fraction			PC1 axis scores			PC2 axis scores		
	1	2	3	1	2	3	1	2	3	1	*2*	3
All vertically transmitted	**↑**	–	ns	**↓**	–	**↓**	–	ns	ns	–	ns	ns
All horizontally transmitted	**↓**	–	**↓**	ns	–	**↓**	–	ns	ns	–	ns	ns
*Borrelia burgdorferi* s.l.(H)	**↓**	–	**↓**	ns	–	ns	–	ns	ns	–	**↑**	**↑**
*Anaplasma phagocytophilum*( H)	**↑**	–	ns	ns	–	ns	–	ns	ns	–	ns	ns
*Neoehrlichia mikurensis* (H)	ns	–	ns	ns	–	↓	–	ns	ns	–	ns	ns
*Babesia microti* (H)	ns	–	ns	ns	–	ns	–	ns	ns	–	ns	ns
*Midichloria mitochondrii* (V)	**↑**	–	ns	**↓**	–	ns	–	ns	ns	–	ns	ns
*Borrelia miyamotoi* (V)	ns	–	ns	ns	–	ns	–	**↓**	ns	–	ns	ns
*Rickettsiella* spp. (V)	ns	–	ns	ns	–	ns	–	↓	ns	–	ns	ns
*Babesia* spp. (V)	ns	–	ns	ns	–	ns	–	ns	ns	–	ns	ns
*Rickettsia helvetica* (V)	ns	–	ns	ns	–	ns	–	ns	ns	–	ns	ns
*Spiroplasma ixodetis* (V)	ns	–	ns	↑(ns)	–	ns	–	ns	ns	–	ns	ns

↑ Positive associations; ↓ negative associations; ns (not significant association)

Experiment 1: tick physiology. Experiment 2: tick behaviour. Experiment 3: tick physiology and behaviour

PC1 and PC2 axis scores refer to the principal components that were positively correlated with the questing height (average questing height and maximum questing height) and time spent in the low humidity zone (PC1) or positively associated with walking distance and velocity and to a lesser extent with the high humidity zone (PC2)

## Discussion

Our data show that tick symbionts are differentially associated with tick physiology and behaviour. We hypothesized that we would detect positive correlations between the presence of horizontally transmitted symbionts and tick activity and between vertically transmitted symbionts and tick weight and lipid fraction. Our data showed indeed that horizontally transmitted symbionts were associated with higher activity and that vertically transmitted symbionts were associated with a larger body weight. In contrast, our hypothesis concerning the lipid fraction was rejected because the presence of vertically transmitted symbionts was associated with a lower lipid fraction in the ticks. The strongest associations with tick physiology and behaviour were observed for *B. burgdorferi* s.l. and *M. mitochondrii*. As these two symbionts were the most prevalent species in our field-collected ticks, they may have driven the overall effects observed in the analysis of pooled horizontally and vertically transmitted symbionts. Of note, the date at which the ticks were collected was significantly associated with tick physiology and behaviour. This may be explained by the micro-climatic conditions or the ticks’ age at the time of collection. While controlling for the effects of ‘date of collection’ (by including them into our statistical models), we demonstrated that specific symbionts were still significantly associated with tick physiology and tick weight.

The horizontally transmitted *B. burgdorferi* s.l. was associated with increased tick activity and lower body weight. Associations between *B. burgdorferi* s.l. and tick behaviour or physiology have been observed in earlier studies, but results are frequently contradictory [14]. While some studies found increased activity of *B. burgdorferi* s.l. infected ticks [31, 48], others identified a negative effect on tick activity [49, 50]. Furthermore, in contrast to our results, multiple studies have observed a positive relation between tick weight and *B. burgdorferi* s.l. infection [51, 52]. We found a negative association of *B. burgdorferi* s.l. infection with tick weight in two independent experiments, indicating the consistency of our findings. The negative associations of *B. burgdorferi* s.l. with tick weight may be the result of increased activity, reducing the weight of ticks as a consequence of the increased metabolic conversion of lipids, glycogen and carbohydrates [6, 7]. Nevertheless, the current study did not detect differences in lipid fractions of *B. burgdorferi* s.l. infected ticks, in contrast to other studies (reviewed in [53]). While we used an adapted lipid determination protocol, both the dry and lipid weighs of ticks obtained in this study were in accordance with previously published literature (Additional file 1: Fig. S2) [6, 50, 51, 54]. Methodological differences are therefore unlikely to explain the inconsistencies with previous studies. The lipid content of ticks is known to vary between months and years as a consequence of tick age and differences in metabolic conversion due to fluctuating climatic conditions [6, 7]. This increases the complexity of understanding the interactions between symbionts and tick physiology and warrants future studies under controlled conditions to understand the mechanisms underlying the observed behavioural and physiological effects of *B. burgdorferi* s.l. on ticks.

The increased activity of *B. burgdorferi* s.l.-infected ticks in the current study may be the result of manipulation by the bacterium to increase the host contact rates of ticks. One of the mechanisms underlying the manipulation of host behaviour is via increased expression of neurotransmitters such as dopamine, octopamine and serotonin [55]. Manipulation of hosts via neural pathways has been observed in multiple arthropods including ants, caterpillars and amphipods [55]. For example, dopamine is known to enhance the locomotion of arthropods [55, 56], and high dopamine levels are reported in parasitised arthropods [57, 58]. Infection with *Borrelia* does increase the response to specific olfactory stimuli [59]; however, whether this difference in behaviour is related to altered neurotransmitter expression remains elusive. In any case, a causal relationship between *B. burgdorferi* s.l. infection and increased activity of ticks seems plausible, as it directly increases the probability of pathogen transmission. Nonetheless, a delicate balance may exist between the strength of this effect and the impact of microclimate: when abiotic conditions are optimal, increased activity increases the probability of acquiring a blood meal, and *B. burgdorferi* s.l. infection may thus enhance tick survival. However, under less suitable conditions, increased questing activity may cause energy reserves to be depleted before a host is encountered, resulting in death of the tick vector and hence in the end of the transmission cycle. Future studies should address this question in more detail.

While horizontally transmitted symbionts such as *B. burgdorferi* s.l. rely on a vertebrate host for their transmission, vertically transmitted symbionts rely primarily on their invertebrate host for survival and proliferation. We observed a high infection rate of vertically transmitted symbionts (73%), which suggests some form of mutualistic relationship with ticks [12]. In the current study, infection with the vertically transmitted *M. mitochondrii* was associated with higher body weight. The higher weight of ticks favours their survival since a larger body size increases water retention [51], making larger ticks less prone to desiccation. In addition, vertically transmitted symbionts co-evolved with their host [3, 60], most likely as they provide ticks with essential nutrients such as vitamins that are absent in the haematophagous diet of ticks [12]. For example, *M. mitochondrii* possesses pathways for the biosynthesis of B vitamins [61]. These B vitamins are essential for the growth and survival of ticks [16]. In addition, the *M. mitochondrii* genome encodes a cytochrome cbb3 oxidase that enables the synthesis of ATP at low oxygen concentrations [62, 63], which is hypothesized to occur during blood feeding and oogenesis [64, 65]. Hence, the presence of *M. mitochondrii* may also enhance tick weight and survival via this route.

Another explanation for the higher weight of *M. mitochondrii*-infected ticks is a possible difference in sex ratio between *M. mitochondrii*-infected and -uninfected nymphs. This is observed in adult ticks, where female infection rates of *M. mitochondrii* are close to 100%, while only a fraction of male ticks are infected [27, 66]. Furthermore, adult female ticks are heavier compared to male ticks and engorged female nymphs are heavier compared to engorged male nymphs [66]. It is however not known whether this weight difference between sexes is already present in the larval or unfed nymphal stages that were used in the current study. We could therefore not confirm whether weight differences in the nymphal stage between *M. mitochondrii*-infected and uninfected ticks were in fact the result of sex differences.

Besides *B. burgdorferi* s.l. and *M. mitochondrii*, other tick symbiont species had a positive association with tick weight, such as *A. phagocytophilum*, or with PC1 axis scores (i.e. tick questing height), such as *B. miyamotoi* and *Rickettsiella* spp. Interestingly, whereas *B. burgdorferi* s.l.-infected ticks had a lower body weight, *A. phagocytophilum*-infected ticks had a higher body weight. *Anaplasma phagocytophilum* circulates primarily in a tick-deer cycle [34], while *B. burgdorferi* s.l. primarily circulates in a tick-bird or tick-rodent cycle [67]. Ticks that feed on deer may take larger blood meal sizes compared to ticks feeding on birds or rodents, which could explain the larger body weights of ticks infected with *A. phagocytophilum*. Nevertheless, this hypothesis has not been tested to the best of our knowledge.

The presence of multiple symbiont species infecting ticks at the same time is frequently observed [1] and in the current study 24.2% of the nymphs were co-infected with at least two symbiont species. Such co-infections can potentially influence tick physiology and behaviour. For example, we observed that ticks infected with *B. burgdorferi* s.l. had a lower body weight, whereas infection with *M. mitochondrii* was associated with higher body weight. Co-infection with both symbionts cancelled out these effects, as co-infected ticks had a similar weight compared to uninfected ticks. Moreover, ticks co-infected with *B. burgdorferi* s.l. and *M. mitochondrii* had higher activity compared to single infections or ticks not infected with any of the two symbiont species. Of note, we only observed this association in one of the two behavioural experiments. Besides, *M. mitochondrii* has been associated with increased replication of *Rickettsia parkeri* in *Amblyomma maculatum* ticks [68]. Moreover, co-infection with *M. mitochondrii* has been linked to increased replication of pathogens in *Amblyomma* ticks, highlighting the importance of understanding the effects of co-infections on tick physiology and behaviour and their potential to promote the transmission of pathogenic symbionts.

As infection of *M. mitochondrii* in ticks was associated with an increase in tick weight, and the infection of *B. burgdorferi* s.l. was associated with an increase in tick activity, these changes in tick physiology and behaviour may increase the transmission of pathogens by increasing host contact rates through prolonged questing activity or increased speed of walking. Furthermore, the higher weight of ticks could affect the size of subsequent blood meals and promote the transmission or ingestion of pathogens [13]. However, it is important to note that symbiont prevalence varies among geographical regions [38], and the coevolution between ticks and specific symbionts is not stable [60]. This suggests that the effects of symbionts on tick weight and activity may also vary in different regions. Additionally, other essential metabolites such as lipids, glycogen, proteins and carbohydrates also play a role in tick physiology [7] and may be influenced by the presence of symbionts. Therefore, further investigation under controlled conditions is needed to fully understand the underlying mechanisms of symbiont-induced effects on tick physiology and behaviour.

## Conclusions

In conclusion, our study provides new insights into the associations between tick symbionts and tick physiology and behaviour. We showed that ticks infected with vertically transmitted symbiont species such as *M. mitochondrii* have a higher body weight, whereas ticks infected with horizontally transmitted symbiont species *B. burgdorferi* s.l. have a lower body weight. These symbionts may influence their transmission by manipulating the behaviour and physiology of ticks. This calls for controlled infection studies with selected symbiont species in naïve ticks to elucidate these symbiont-tick associations on an individual level to improve our understanding of the variation in infection risk of tick-borne pathogens.

### Supplementary Information

**Additional file 1: Figure S1.** Lipid and weight of ticks. **Figure S2.** Effect of temperature incubation on tick weight and lipid fraction after 14 weeks at either 6 °C or 30 °C. **Figure S3.** Tick activity set-up (**A**) and saturation deficit gradient inside the behavioural assay (**B**). **Table S1.** Prevalence (%) of tick symbiont co-infections in *Ixodes ricinus* nymphs of all experiments combined. **Table S2.** Results of GLMs of symbiont effects on the PC1 and PC2 axis scores. **Figure S4.** Histograms of tick weight, lipid weight and lipid fraction in *Ixodes ricinus* nymphs.

## Data Availability

The datasets used and/or analysed during the current study are available in the supplementary data files and from the corresponding author on reasonable request.
