# Supplementary material for: Differential associations of horizontally and vertically transmitted symbionts on Ixodes ricinus behaviour and physiology
Source: Parasit Vectors. 2023 Nov 29;16:443. doi: 10.1186/s13071-023-06025-3 (PMC10685571; doi:10.1186/s13071-023-06025-3)
Supplement: Supplementary file 1 — Additional file 1: Figure S1. Lipid and weight of ticks. Figure S2. Effect of temperature incubation on tick weight and lipid fraction after 14 weeks at either 6 °C or 30 °C. Figure S3. Tick activity set-up (A) and saturation deficit gradient inside the behavioural assay (B). Table S1. Prevalence (%) of tick symbiont co-infections in Ixodes ricinus nymphs of all experiments combined. Table S2. Results of GLMs of symbiont effects on the PC1 and PC2 axis scores. Figure S4. Histograms of tick weight, lipid weight and lipid fraction in Ixodes ricinus nymphs. [file 13071_2023_6025_MOESM1_ESM.docx]

**Additional file data**


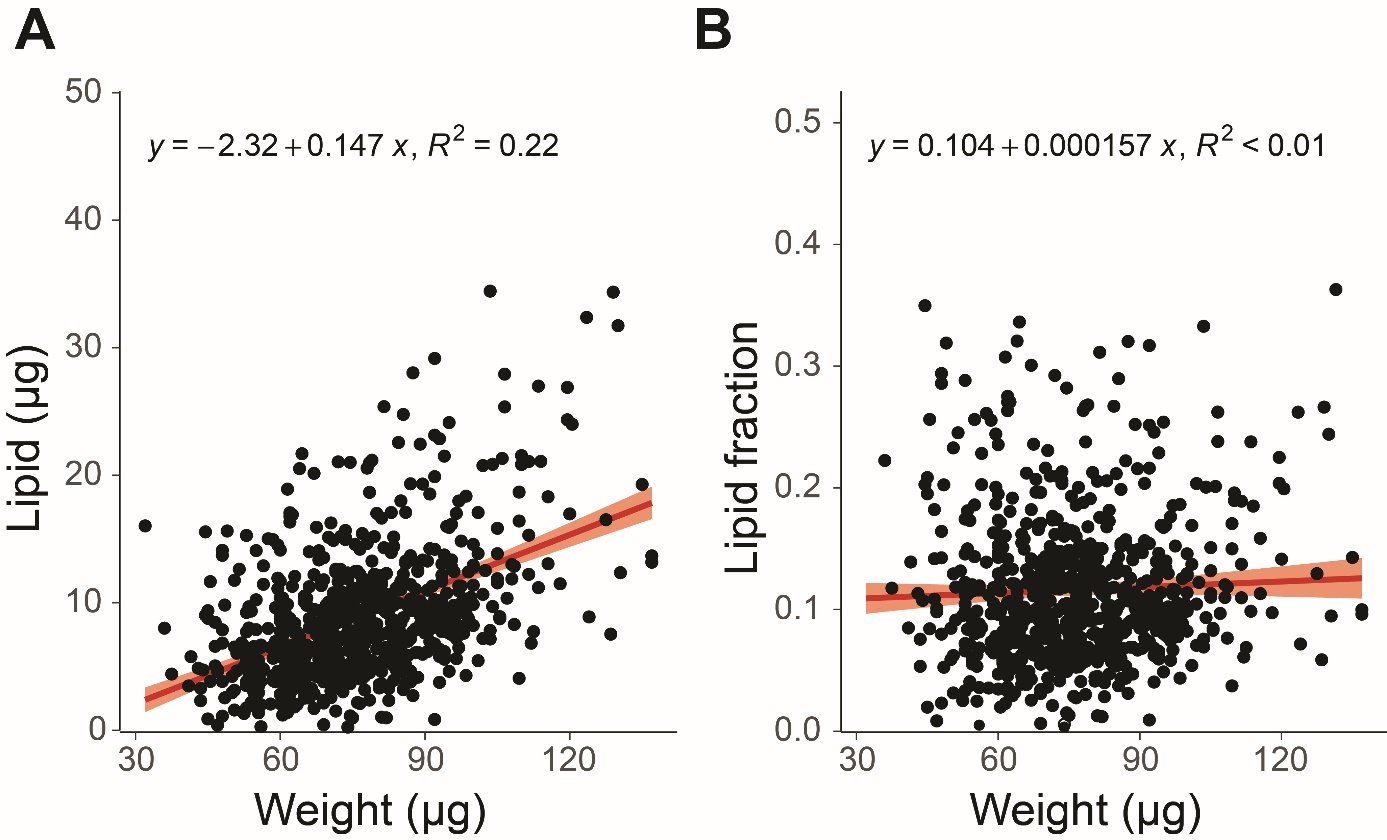


**Figure S1. Lipid and weight of ticks.** A) Lipid content increases with the size of the ticks. B) Correcting the lipid content of ticks for tick dry weight gives the lipid fraction.


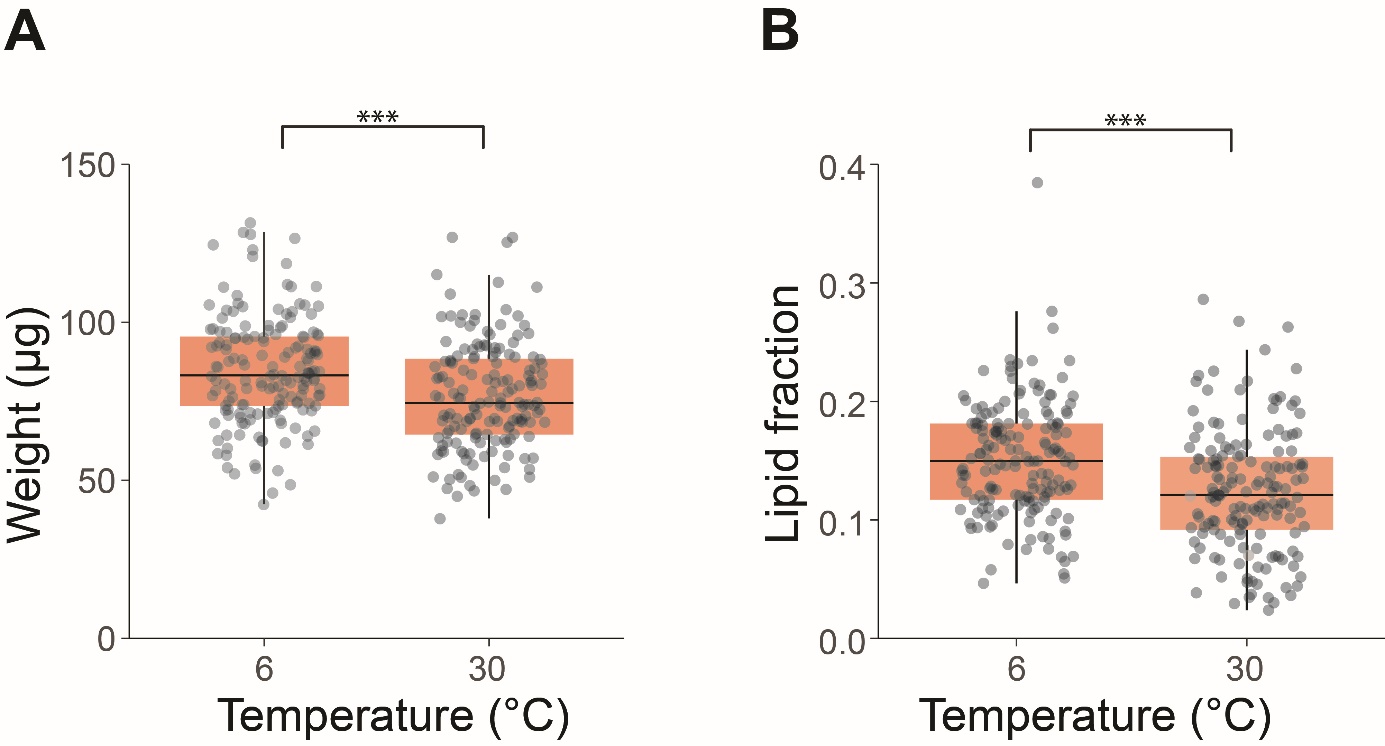


**Figure S2.** **Effect of temperature incubation on tick weight and lipid fraction after 14 weeks at either 6**º**C or 30**º**C.** Field-collected nymphs were incubated at either 6ºC (n = 165) or 30ºC (n = 161) for 14 days. Tick weight was measured twice and the average dry weight was used for analysis. Ticks incubated at 30ºC had a significantly lower body weight (GLM, LRT, χ^2^ = 19.4, df = 1, p < 0.001) and significantly lower lipid fraction (GLM, LRT, χ^2^ = 32.3, df = 1, p < 0.001) compared to ticks incubated at 6ºC.

**
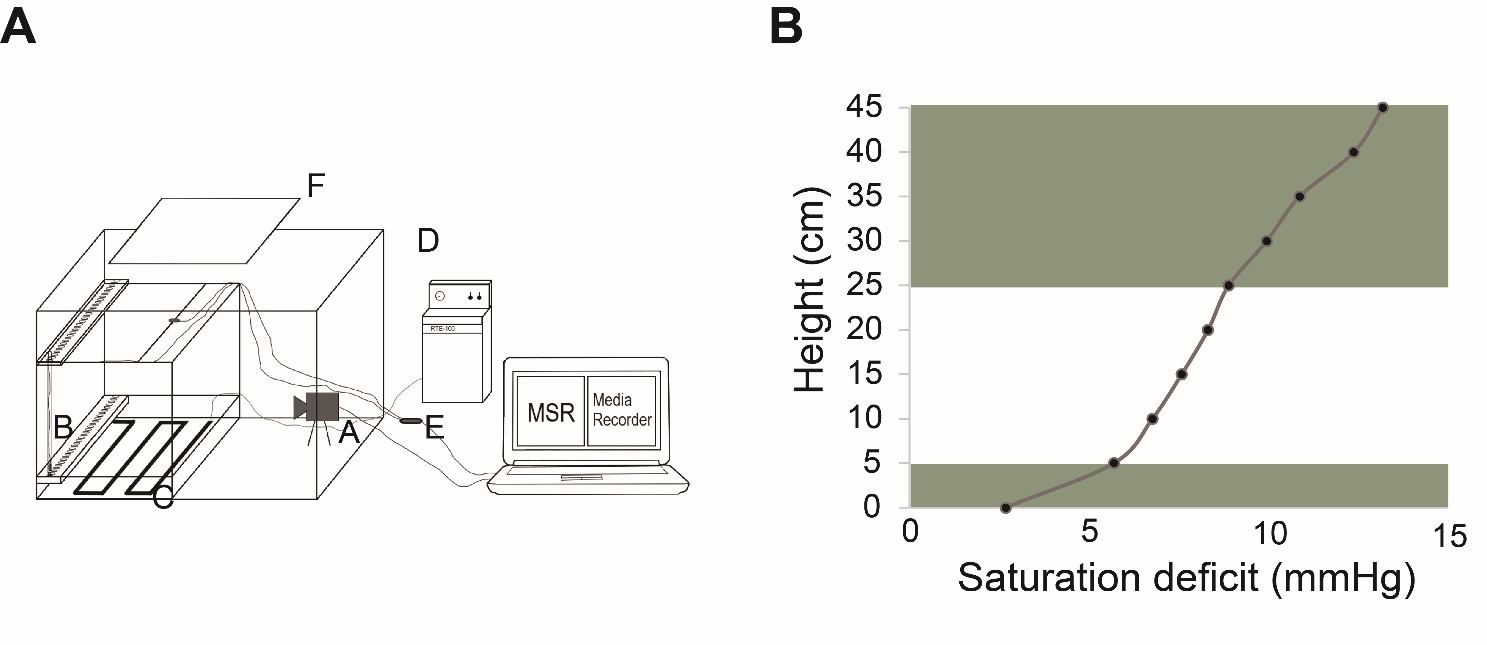
**

**Figure S3. Tick activity set-up (A) and saturation deficit gradient inside the behavioural assay (B). A)** A Basler acA1920-155um camera (A, Basler, Ahrensburg, Germany) was used to record the behaviour of ticks in vertically positioned tubes (B). The bottom of the tubes was in contact with a 10 °C water layer, which was kept at a constant temperature by a copper structure (C) across the bottom that was connected to a water bath circulator (D). With data loggers that were connected to a laptop. The temperature and the relative humidity were determined at the start and the end of a run at the bottom of an empty tube and in the environment (E). The setup was constantly illuminated by two LED panels during each run (F). **B)** The height represents the height in the transparent polycarbonate tubes of the behavioural assay. The saturation deficit (SD, in mmHg) was calculated according to Perret et al. (2000) and measured in the tubes using an MSR datalogger with external probes. Green shading represents the high humidity zone (SD < 5.6, between 0 and 5 cm) and the low humidity zone (SD > 9, between 25 and 45 cm).

**Table S1.** Prevalence (%) of tick symbiont co-infections in *I. ricinus* nymphs of all experiments combined.

|  | *B. burgdorferi* s.l. | *B. miyamotoi* | *A. phagocytophilum* | *N. mikurensis* | *S. ixodetis* | *R. helvetica* | *M. mitochondrii* | *Rickettsiella spp.* | *Babesia spp.* |
| --- | --- | --- | --- | --- | --- | --- | --- | --- | --- |
| *B. miyamotoi* | 0.6% |  |  |  |  |  |  |  |  |
| *A. phagocytophilum* | 0.5% | 0.1% |  |  |  |  |  |  |  |
| *N. mikurensis* | 2.3% | 0.4% | 0.3% |  |  |  |  |  |  |
| *S. ixodetis* | 1.9% | 0.3% | 0.2% | 1.1% |  |  |  |  |  |
| *R. helvetica* | 1.4% | 0.2% | 0.2% | 0.8% | 0.7% |  |  |  |  |
| *M. mitochondrii* | 12.9% | 2.1% | 1.5% | 7.5% | 6.3% | 4.5% |  |  |  |
| *Rickettsiella spp.* | 0.9% | 0.1% | 0.1% | 0.5% | 0.4% | 0.3% | 3.0% |  |  |
| *Babesia spp.* | 0.4% | 0.1% | 0.0% | 0.2% | 0.2% | 0.1% | 1.3% | 0.1% |  |
| *B. microti* | 0.1% | 0.0% | 0.0% | 0.0% | 0.0% | 0.0% | 0.2% | 0.0% | 0.0% |

**Table S2.** Results of GLMs of symbiont effects on the PC1 and PC2 axis scores.

| **Variable** | **Model type** | **Model** | **Factor** | **df** | **χ^2^** | **p** |
| --- | --- | --- | --- | --- | --- | --- |
| PC1 axis scores | GLM (Gamma) | ~Horizontally +Vertically+ Collection date + Time of the assay | Horizontal | 1 | 2.364 | 0.124 |
|  |  |  | Vertical | 1 | 1.894 | 0.168 |
|  |  |  | Date | 4 | 35.902 | < 0.001 |
|  |  |  | Time | 1 | 9.439 | < 0.001 |
| PC2 axis scores | GLM (Gamma) | ~Horizontally +Vertically+ Collection date + Time of the assay | Horizontal | 1 | 1.612 | 0.204 |
|  |  |  | Vertical | 1 | 0.001 | 0.974 |
|  |  |  | Date | 4 | 43.762 | < 0.001 |
|  |  |  | Time | 1 | 22.803 | < 0.001 |

**
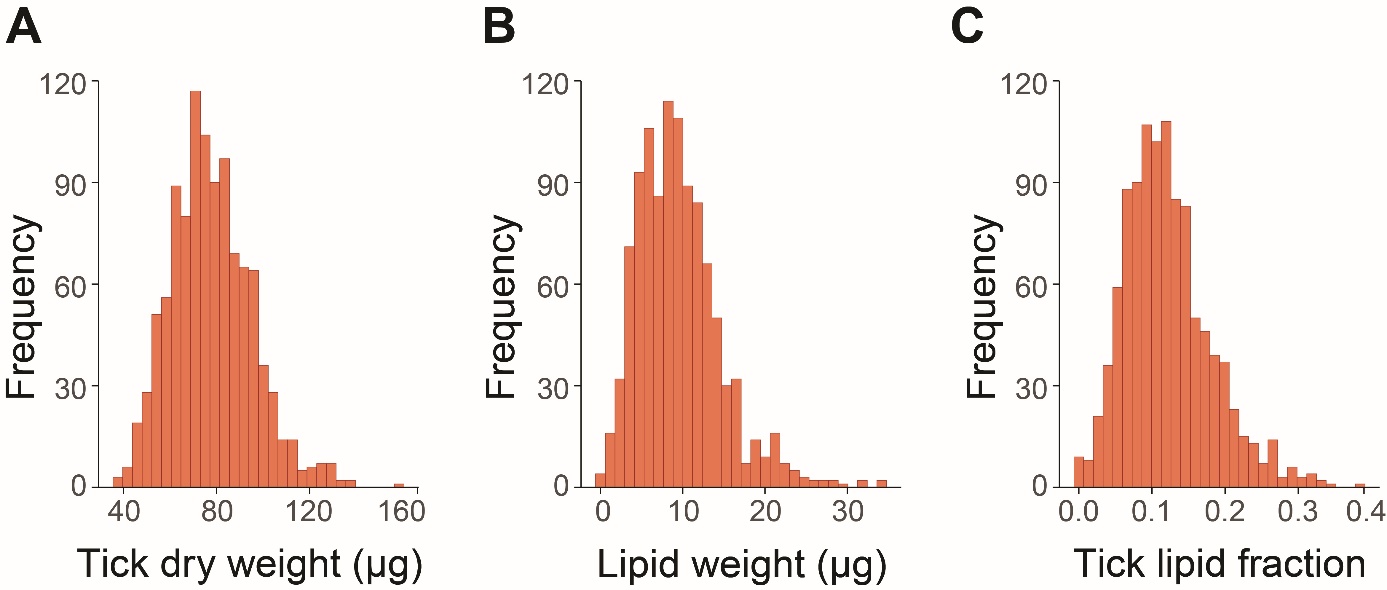
**

**Figure S4.** Histograms of (A) tick weight, (B) lipid weight and (C) lipid fraction in *I. ricinus* nymphs. The lipid fraction was calculated by dividing the lipid weight by the tick dry weight. Frequency expressed as number of ticks.
